# Influence of Drilling Protocol on Primary Implant Stability Depending on Different Bone Qualities and Implant Macro-Designs, Lengths, and Diameters

**DOI:** 10.3390/jfb16080296

**Published:** 2025-08-16

**Authors:** Milan Stoilov, Ramin Shafaghi, Lea Stoilov, Helmut Stark, Michael Marder, Norbert Enkling, Dominik Kraus

**Affiliations:** 1 Department of Prosthodontics, Preclinical Education and Dental Materials Science, University Hospital Bonn, 53111 Bonn, Germany; stoilov@uni-bonn.de (M.S.); lea.stoilov@ukbonn.de (L.S.); helmut.stark@uni-bonn.de (H.S.); michael.marder@ukbonn.de (M.M.); norbert.enkling@unibe.ch (N.E.); 2 Department of Reconstructive Dentistry and Gerodontology, School of Dental Medicine, University of Bern, 3012 Bern, Switzerland

**Keywords:** primary stability, insertion torque, bone quality, drilling protocol, implant geometry

## Abstract

Background: Primary implant stability is a critical factor for successful osseointegration and long-term implant success. This study investigates the impact of drilling protocol modifications on primary stability, considering different bone qualities and implant macro-designs, lengths, and diameters. Material and Methods: Three implant designs—two parallel-walled and one tapered—were tested with diameters ranging from 3.4 to 5.2 mm and lengths from 7.5 to 14.5 mm. Implants were placed in polyurethane foam blocks simulating different bone densities (10, 15, 25, and 35 PCF). A standard drilling protocol was used in all groups, with modifications based on bone quality: overpreparation in dense bone and underpreparation in softer bone. Primary stability was evaluated using insertion torque (IT). The optimal IT range was defined as 25–50 Ncm, based on clinical guidelines for immediate loading. The influence of drilling protocol adaptations on stability parameters was assessed. Results: Insertion torque was primarily influenced by bone density and implant diameter, with implant length playing a minor role. In dense bone (D1, D2), underpreparation improved torque values, especially in smaller implants, while overpreparation reduced them. The highest torques occurred with 5.2 mm implants, sometimes exceeding 80 Ncm. Standard protocols did not consistently achieve optimal torque across implant types. In soft bone (D3), underpreparation—particularly with tapered implants—was modestly beneficial. In very soft bone (D4), none of the protocols reliably reached the desired torque range. Conclusions: Adapting drilling protocols to bone density improves insertion torque, especially with wider implants and in denser bone. Underpreparation is generally more effective than overpreparation. However, in very soft bone, neither implant geometry nor drilling adaptations reliably achieve optimal primary stability, highlighting the need for additional strategies.

## 1. Introduction

An optimal primary implant stability is a crucial prerequisite for successful immediate loading and long-term success [1,2,3,4]. This stability refers to the mechanical interlocking between the implant surface and the surrounding bone immediately after placement [5,6,7]. It is characterized by the frictional force at the implant–bone interface, which prevents implant mobility. Clinically, primary stability is indicated by the absence of implant movement in its final intended position. This is crucial because micro-movements exceeding 150 µm can lead to fibrointegration instead of osseointegration, resulting in implant failure [8].

Primary stability is influenced by multiple factors that collectively determine the mechanical interlocking of the implant within the bone. One key factor is the macro-design of the implant, including its shape and surface characteristics, which significantly impact how well it integrates with the surrounding bone. Additionally, the quality of the bone at the implant site plays a crucial role—denser cortical bone generally provides greater stability compared to softer cancellous bone. The dimensions of the implant, particularly its length and diameter, also affect its ability to achieve a secure fit [9]. Furthermore, the osseous morphology of the surgical site influences the mechanical engagement of the implant, as variations in bone structure can impact stability. Beyond anatomical and implant-related factors, the expertise of the surgeon is another determinant, as precise placement techniques contribute to optimal primary stability. Lastly, the drilling protocol used during site preparation—including the size and type of drills—directly affects the mechanical fit of the implant, further influencing its initial stability.

Traditionally, primary stability was assessed using invasive methods such as measuring insertion and removal torque with a torque gauge or wrench during implant placement. Insertion torque measures the resistance to rotational force during implant placement. Each implant manufacturer typically provides recommended maximum values for insertion torque to ensure optimal primary stability. However, these methods have been largely supplanted by noninvasive techniques like Resonance Frequency Analysis (RFA), which is known for its reliability, repeatability, and correlation with micro-movements [10,11,12]. The RFA system, developed by Meredith in 1996 [13], uses a frequency signal transmitted to a screwed transducer to induce implant vibration. The resulting RFA values are expressed on the Implant Stability Quotient (ISQ) scale, which ranges from 1 to 100. Higher ISQ values indicate greater stability. The RFA system evaluates the stiffness of the bone–implant interface. Despite the lack of consensus on a gold standard evaluation system, both insertion torque assessment and RFA are widely used in clinical practice and research to measure implant stability.

Primary stability is essential for osseointegration, facilitating bone formation around the implant and establishing secondary or biological stability [14,15]. For the prolonged success of immediate or early loading implants, achieving high primary stability is paramount. Clinical factors such as bone quality significantly influence implant survival and osseointegration success [16,17,18]. Poor bone quality (types III and IV according to Lekholm and Zarb [16]) presents challenges due to low-density cancellous bone and thin cortical layers, which can reduce the success rate of immediate implants or loading protocols. Therefore, surgeons often adapt their techniques based on experience to enhance primary stability. Optimal surgical techniques for dental implant placement are predicated on several critical factors, including meticulous osteotomy drill preparation, ensuring initial implant stability, and avoiding excessive heat generation or compressive trauma during the procedure. Determining the appropriate final osteotomy dimension involves adapted drilling techniques that can create varying degrees of bone compression and primary stability, tailored to the specific characteristics of the bone at the implant site. Various surgical modifications have been proposed to improve stability in low-density bone areas. Undersized drilling aims to enhance primary stability. Certain studies recommend the use of undersized preparation or reducing drill dimensions, which involves using a final drill with a smaller diameter than typically recommended [19,20,21]. This approach seeks to locally enhance bone density, thereby improving primary stability, particularly in areas with low-quality bone. One technique involves bone condensation, where condensers are used after the pilot drill to laterally displace bone, increasing its density. Another method is bi-cortical fixation, which engages both cortical plates of the bone to provide greater mechanical support. Additional strategies include preparing the implant bed without profiling and tapping, though outcomes regarding bone-to-implant contact have been variable. These techniques continue to be evaluated and refined to optimize the mechanical stability of dental implants in challenging bone conditions.

Based on our previous report [22] where we determined primary stability of three different macro-geometries of dental implants depending on implant length, diameter, and bone quality, this study aims to compare various site preparation techniques and assess their impact on implant stability, considering the insertion torque (IT) values.

The null hypothesis (H_0_) posits that modifications to the drilling protocol, implant geometry, length, or diameter do not significantly affect primary implant stability as measured by insertion torque.

## 2. Materials and Methods

In this study, three different implant designs, each of different lengths and diameters (all from SIC invent^©^, Basel, Switzerland; Table 1), were placed in solid rigid polyurethane foam blocks (Sawbones, Vashon, WA, USA) with four different densities (10 PCF, 15 PCF, 25 PCF, 35 PCF; PCF = pounds per cubic foot). Depending on the density of the artificial bone blocks, different implant-side preparations (undersized and oversized drilling) were carried out prior to implant placement (Table 2). Primary implant stability was measured by insertion torque (IT).

### 2.1. Implants Characteristics

All implants employed in this study were conditioned through a blasting process with zirconia beads, followed by acid cleaning (SICmatrix^®^). This treatment produced a surface with moderate roughness (Sa = 1.0 µm). The research assessed the performance of three different dental implant macro-designs with two cylindrical implants (SICace^®^ and SICmax^®^) and one tapered implant (SICtapered^®^). Further information on the properties of the different implant types can be found in [22].

### 2.2. Implant Site Preparation

**Figure 1:**
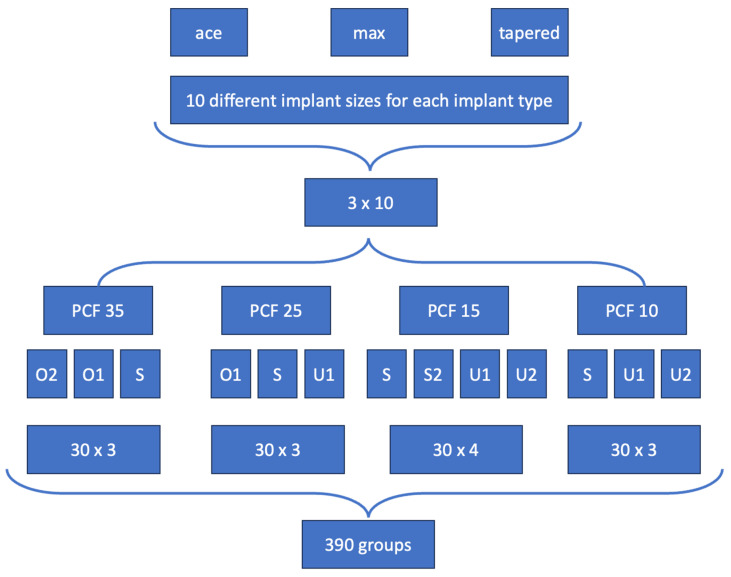
Schematic illustration of the implant site preparations and study design. A total of 390 groups were evaluated. In each group, six implant sites were prepared, resulting in a total of 2.340 drillings. The implants used were SICace^®^ (ace), SICtapered^®^ (tapered), and SICmax^®^ (max). The definitions of the abbreviations O1, O2, S, S2, U1, and U2 are provided in Table 2.

A CAD drawing was created to evenly transfer a matrix of rows and columns onto the various polyurethane foam blocks to prepare the implant sites. Six implants were used for each group (n = 6), resulting in a total of 2.340 implant holes for each simulated bone density (Figure 1). The drilling was executed using a high-precision, CNC-controlled milling machine (Pro^©^, Mekanika, Anderlecht, Belgium). Original SIC drills were used, following the SIC drilling protocol with the recommended drilling speed of 300 rpm. This method ensured that the implant osteotomies precisely matched the ideal contours of the SIC drills, eliminating manual errors such as angle deviations or incorrect drilling depths. The various implant types, diameters, lengths, and drilling sequences (final drill and crestal drill) are detailed in Table 1 and Table 2.

### 2.3. Implant Insertion and Measurement of Implant Stability

**Figure 2:**
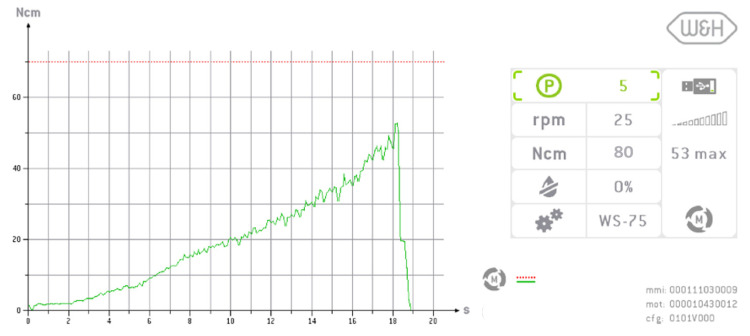
Dynamic measurement of insertion torque (IT) during implant placement using a surgical motor. The development of IT over time and with increasing implant depth within the osteotomy site is clearly visible. In this case, a maximum torque of 53 Ncm was recorded.

All implants were inserted into the prepared sites using a torque-controlled surgical motor unit and a contra-angle handpiece (Implantmed Plus and WS-75L; W&H Deutschland GmbH, Laufen, Germany) at 25 rpm by an experienced implantologist. The surgical motor unit recorded the insertion torque throughout the process, with a maximum torque capacity of 80 Ncm (Figure 2). For analysis, the peak insertion torque value for each implant was recorded for statistical evaluation. If the maximum torque of 80 Ncm was reached and the implant shoulder did not align flush with the polyurethane foam block, the protruding length of the implant was measured in millimeters.

### 2.4. Statistical Analysis

Mean maximum insertion torque (IT) values ± standard deviation (SD) were calculated for each group. Each experimental group consisted of *n* = 6 samples, resulting in a total sample size of *N* = 2340. To assess clinical relevance with respect to primary implant stability, an optimal IT range of 25–50 Ncm was defined. Values below 25 Ncm and above 50 Ncm were classified as critical.

**Figure 3:**
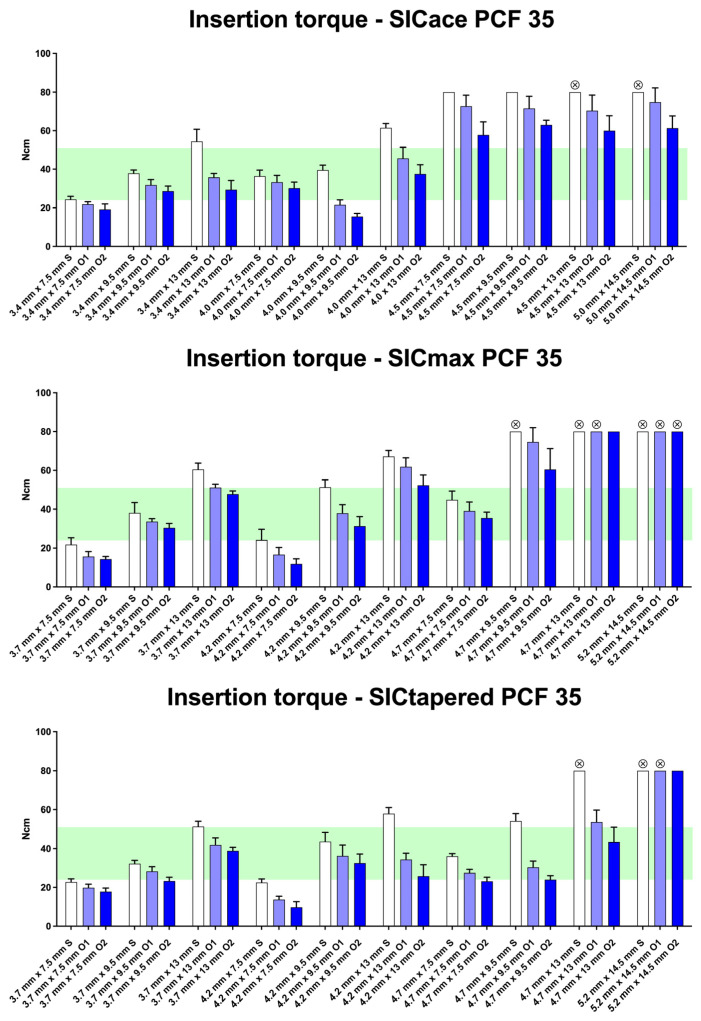
Insertion torque values (mean ± SD) for SICace^®^, SICmax^®^, and SICtapered^®^ implants in polyurethane bone blocks with a density of PCF 35 (representing D1 bone quality). Standard drilling protocols (S) (white bars), and overpreparation protocols O1 and O2 (blue bars) were applied across different implant diameters and lengths. The green band marks the predefined optimal torque range (25–30 Ncm) for achieving good primary stability. The data show that overpreparation generally reduces insertion torque, while some larger-diameter implants exceeded 80 Ncm, triggering a motor stop. Differences between implant designs were minor; notably, parallel-walled implants (SICace^®^, SICmax^®^) sometimes produced higher torque than tapered designs (SICtapered^®^). Symbols at the top of the bars indicate that implants could not be inserted to their full length when the maximum torque of 80 Ncm was reached.

**Figure 4:**
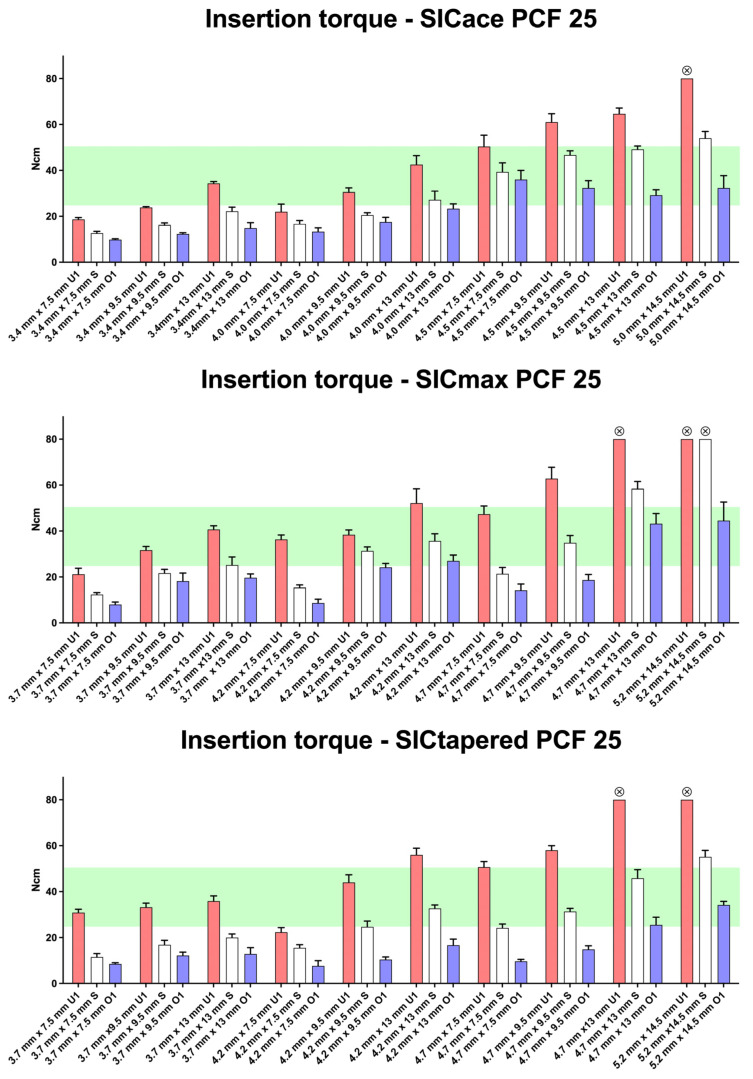
Insertion torque values (mean ± SD) for SICace^®^, SICmax^®^, and SICtapered^®^ implants in polyurethane foam blocks with a density of PCF 25, representing dense bone (D2). Shown are results from standard preparation protocols (white bars), underpreparation protocols U1 (red bars), and overpreparation protocol O1 (blue bars), applied across various implant lengths and diameters. The green shaded area indicates the target range for optimal primary stability (25–30 Ncm). Underpreparation protocols generally increased insertion torque values, particularly for implants with larger diameters. Overpreparation led to a reduction in torque, but in some cases (e.g., 5.2 mm implants), it allowed for torque values to fall within the optimal range. Differences between implant systems were minor, though the SICtapered^®^ design showed slightly elevated torque values compared to parallel-walled designs in several configurations. Symbols at the top of the bars indicate that implants could not be inserted to their full length when the maximum torque of 80 Ncm was reached.

For comparison of the three implant macro designs across different bone qualities, a descriptive statistical analysis was performed, focusing on the frequency distribution of IT values relative to the defined thresholds. To test the overall influence of the preparation protocol in different bone densities, linear mixed models with the implant specifications as random factor, implant design (SICace^®^; SICmax^®^; SICtapered^®^) and preparation protocol as fixed factors were used for each bone density. If necessary, insertion torque (primary outcome) was log-transformed to meet the homoscedasticity assumption. If the insertion torque reached the maximum torque capacity of the surgical motor before the implant was fully seated in the bone, the values for these implants were excluded from the statistical analysis. These groups have been marked accordingly in Figure 3 and Figure 4. All graphical representations were generated using Prism 9 (GraphPad Soft-ware^®^, San Diego, CA, USA). Analysis was performed with SPSS (Version 29.0, IBM, Armonk, NY, USA). All tests were performed at a significance level of α = 0.05. Effect estimates were calculated by Satterthwaite approximation. For multiple comparisons, Bonferroni was used. Values are noted as estimated mean [95% confidence intervals].

## 3. Results

As expected, the achieved insertion torque strongly depends on bone quality, implant geometry, as well as implant length and diameter. Notably, for the tested implant systems, higher torque values can be achieved with larger diameters, and to a slightly lesser extent with longer implants.

**Figure 5:**
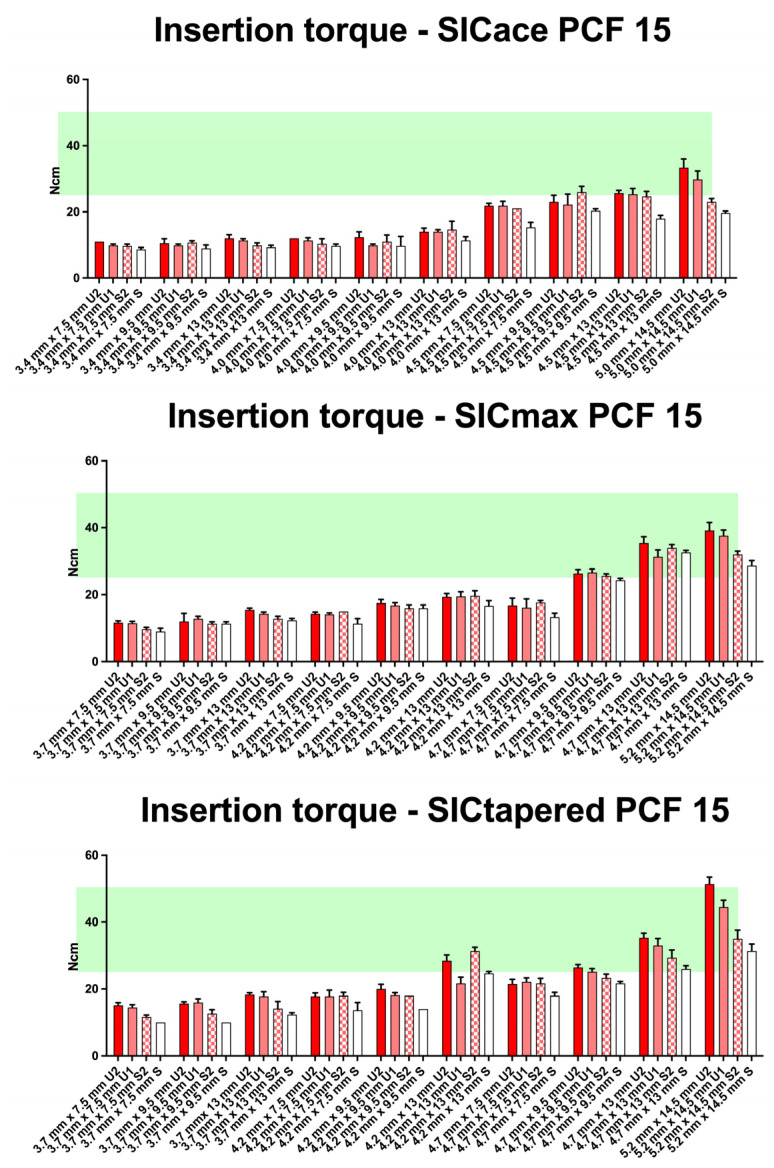
Insertion torque values (mean ± SD) for SICace^®^, SICmax^®^, and SICtapered^®^ implants in polyurethane foam blocks with a density of PCF 15, simulating soft bone conditions (D3). Displayed are outcomes for standard drilling protocols (white bars), underpreparation protocols U1 and U2 (light and solid red bars, respectively), and crestal drill omission (checkered red bars). The green shaded zone represents the defined target range for optimal primary stability (25–30 Ncm). With this bone quality, only select combinations of implant design, diameter, and underpreparation reached the optimal torque window. Undersized osteotomies led to slight increases in insertion torque, particularly with wider implants. The SICtapered^®^ design generally achieved higher torque values than parallel-walled implants under the same conditions, suggesting a potential biomechanical advantage in softer bone.

**Figure 6:**
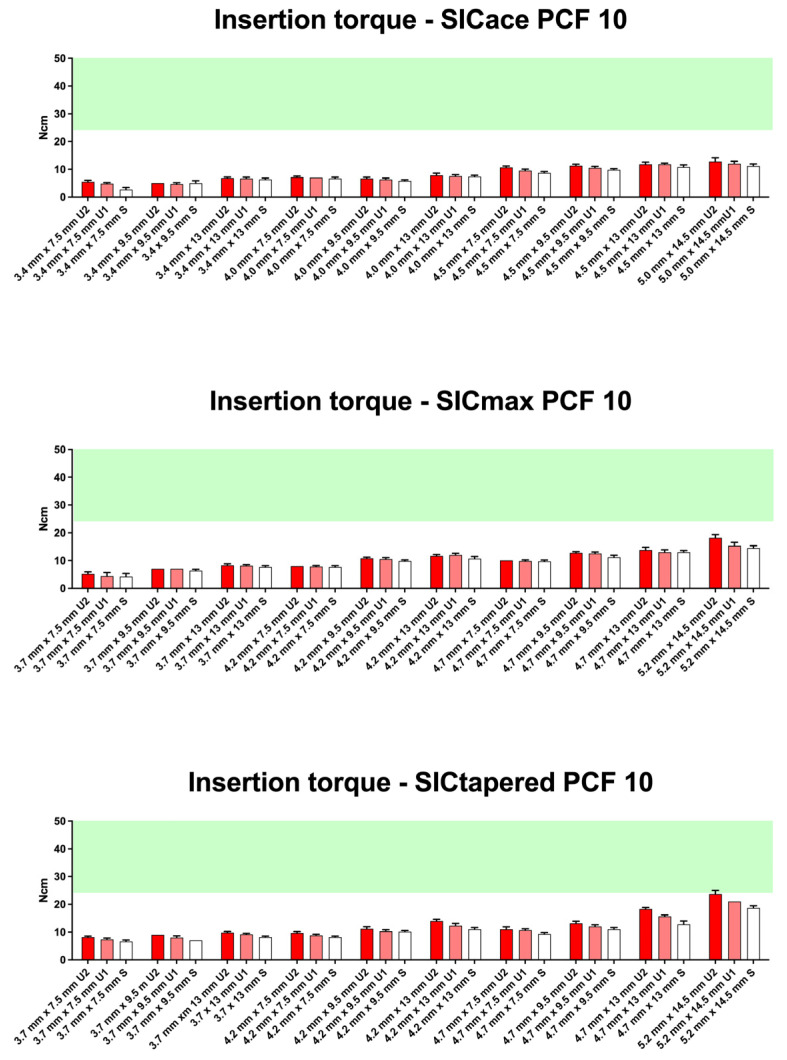
Insertion torque values (mean ± SD) for SICace^®^, SICmax^®^, and SICtapered^®^ implants in polyurethane foam blocks with a density of PCF 10, representing very soft bone (D4). Shown are results for standard drilling protocols (white bars) and underpreparation strategies U1 and U2 (red bars). The green shaded region marks the desired primary stability range (25–30 Ncm). None of the tested implant types or protocol modifications succeeded in consistently reaching the torque threshold for adequate primary stability. Even with underpreparation and tapered macro-designs, insertion torque remained below the desired range, highlighting the limitations of surgical protocol adaptation in extremely soft bone.

As demonstrated in the initial publication [22], the standard preparation protocol does not consistently result in optimal insertion torque (25–30 Ncm) across different bone qualities (D1 to D4) and implant geometries (Figure 3, Figure 4, Figure 5 and Figure 6). Therefore, this follow-up study aimed to investigate whether adapting the drilling protocol to the corresponding bone quality—overpreparation in dense bone and underpreparation in soft bone—could improve the likelihood of achieving the predefined torque range associated with good primary stability. Overall, in all four bone qualities, the adapted drilling protocol had a highly significant effect on the insertion torque (PCF 35: F1, 398 = 115.5; *p* < 0.001; PCF 25: F2, 453 = 850; *p* < 0.001; PCF 15: F2, 671 = 924; *p* < 0.001; PCF10: F2, 485 = 241; *p* < 0.001). Undersized drilling protocols resulted in higher insertion torque values, whereas oversized protocols yielded lower values compared to the standard approach.

This effect was evident in soft bone (D3), where optimal torque was achieved with the SICtapered implant in larger diameters. The influence of the adapted protocol was more pronounced in wider and longer implants compared to smaller sizes. However, in extremely soft bone (D4), modifying the drilling protocol appeared to have little to no meaningful impact on torque values.

In very dense bone (D1), 4 out of 10 SICace^®^ implants achieved the optimal insertion torque with the standard protocol, and similarly 4 out of 10 when using overpreparation protocols O1 or O2 (Figure 3). For SICmax^®^ implants, 3 out of 10 reached the optimal torque with the standard preparation, while 7 out of 10 achieved it following modification of the drilling protocol (Figure 3). The SICtapered^®^ implants showed similar results, with 4 out of 10 reaching the optimal torque under standard preparation and 7 out of 10 after applying overpreparation protocols (O1 and O2).

Overpreparation of the implant site resulted in a reduction in insertion torque values across all groups. Exceptions were observed in the SICmax^®^ and SICtapered^®^ groups with the largest implant diameter (5.2 mm), where insertion torque values consistently exceeded 80 Ncm, leading to an interruption of the procedure by the implant motor. The macro-geometry of the implants appeared to play only a minor role in D4 bone density. The parallel-walled implants (SICace^®^ and SICmax^®^) showed similar or even higher torque values compared to the tapered (SICtapered^®^) implants. This was evident when comparing SICmax^®^ to SICtapered^®^, where SICmax^®^ tended to show higher insertion torque under standard preparation.

In dense bone (D2), 4 out of 10 SICace^®^ implants achieved the specified torque range using the standard preparation protocol (Figure 4). The same result (4/10) was observed when using either underpreparation (U1) or overpreparation (O1) protocols. For SICmax^®^ implants, 4 out of 10 also reached the target torque with the standard protocol, 5 out of 10 with underpreparation (U1), and 3 out of 10 with overpreparation (O1). However, when focusing on implants with smaller diameters (3.7 mm, 4.0 mm, and 4.2 mm), it becomes evident that achieving the required torque threshold of >25 Ncm in dense bone is sometimes only possible when using the underpreparation protocol (U1). The implant geometry of the three implant types appeared to have a comparable influence on insertion torque. Overpreparation (O1) consistently led to a reduction in torque values across all groups compared to the standard protocol. This protocol, however, allowed for large implants (e.g., 5.2 mm × 14.5 mm) to reach insertion torque values within a range physiologically acceptable for the bone.

In soft bone (D3), four different preparation protocols were applied. None of the SICace^®^ implants (0/10) reached the required torque range for immediate loading protocols when using the standard protocol (Figure 5). When the crestal drill was omitted during site preparation, one implant reached the target range. Underpreparation protocols U1 and U2 led to two implants each achieving insertion torques between >25 Ncm and <50 Ncm. For SICmax^®^ implants, 2 out of 10 reached the required torque threshold with the standard protocol. The modified drilling protocols (S2, U1, and U2) resulted in 3 out of 10 implants each achieving adequate primary stability. A positive correlation between increasing implant diameter and insertion torque was observed in this group as well. However, unlike in D1 and D2 bone, no consistent relationship between implant length and torque increase could be demonstrated within the respective diameter-specific groups. Omitting the crestal drill (S2) led to only a limited improvement in primary stability, observed in just three subgroups (SICace^®^: 4.5 mm × 9.5 mm, SICmax^®^: 4.7 mm × 9.5 mm, and SICtapered^®^: 4.2 mm × 13 mm). Underpreparation showed only minimal improvements in primary stability, with the most notable effects seen in implants with larger diameters. The tapered implant geometry tended to generate slightly higher insertion torques in soft bone compared to parallel-walled implant designs; however, this difference was not statistically significant.

In very soft bone (D4), none of the implants reached the required torque range (Figure 6). The effect of a larger implant diameter was minimal in this context. Even underpreparation of the implant site (U1 and U2) led to only a slight increase in insertion torque for the SICmax^®^ and SICtapered^®^ implants.

## 4. Discussion

As previously described, the primary stability of a dental implant at the time of insertion is a crucial factor for subsequent osseointegration [23,24], particularly when immediate loading protocols are pursued [25]. Primary stability is influenced on the one hand by the density and quality of the local bone, and on the other hand by the implant design, including its diameter and length. In our preliminary study, the effect of these implant-specific parameters on various bone qualities was investigated [22]. It was found that larger implant diameters resulted in higher insertion torques in low-density bone, thereby improving primary implant stability; tapered implants were shown to generate particularly high insertion torques in soft bone (compared to parallel-walled designs); and the influence of implant-specific parameters was minimal in very soft bone. This investigation was carried out exclusively using the standard drilling protocol provided by the manufacturer. However, it is well known in clinical practice that modifying the drilling protocol in cases of either high or low bone density can help adjust the insertion torque (IT), thereby achieving the primary stability required for immediate loading. In soft bone (D3), underpreparation protocols are typically employed. These create a situation in which the implant body is slightly larger than the osteotomy, leading to bone condensation and thus enhanced mechanical stability [26]. In contrast, in dense bone (D1), the standard drilling protocol often fails to allow for complete seating of the implant. In such cases, excessively high insertion torques (>80 Ncm) can be reached early during placement, which may result in marginal bone loss around the implant [27].

This follow-up study builds upon our earlier work by evaluating the effects of adapted drilling protocols on primary stability in polyurethane foam blocks simulating different bone densities (10–35 PCF). Using the same implant designs (SICace^®^, SICmax^®^, SICtapered^®^), we now incorporate drilling protocol modifications to address the limitations identified in our initial findings. This study aims to develop evidence-based guidelines for optimizing drilling protocols in clinical practice.

In this study, a standardized in vitro model was employed using rigid polyurethane foam blocks of varying densities to simulate different bone qualities, in accordance with ASTM F1839-08 guidelines [28]. Although such materials do not replicate the biological complexity of human bone, they offer consistent and reproducible mechanical properties, making them suitable for comparative biomechanical analyses. To reflect the clinical scenario of immediate implant placement, single-layer foam blocks were used, where primary stability is primarily derived from apical bone engagement rather than from crestal cortical support [29,30,31]. This experimental setup differs from studies utilizing double-layer configurations to simulate healed ridges with distinct cortical and cancellous components, which typically result in higher insertion torque values [32,33]. By contrast, the monolayer design adopted in both studies represents a more clinically relevant condition for assessing implant stability in fresh extraction sockets. Nevertheless, it must be emphasized that the polyurethane foam model cannot fully reproduce the structural and biochemical intricacies of human trabecular and cortical bone. Therefore, while the results provide valuable comparative biomechanical insights, caution should be exercised when extrapolating these findings directly to clinical outcomes.

To ensure standardized implant site preparation, new drills and a CNC machine were used throughout this study. As recommended by the manufacturer, drills were replaced after every 20 osteotomies. Furthermore, the CNC machine was calibrated daily prior to its first use; however, minor variations cannot be entirely excluded.

In accordance with the literature, a torque range was defined both in this study and in the preceding investigation that appears generally suitable for the immediate loading of a freshly inserted implant. This range for achieving sufficient primary stability is considered to be above 20 Ncm for splinted implants [34]. In the case of single-tooth implants, a higher insertion torque—typically exceeding 30 Ncm, and more specifically >32 Ncm—is generally recommended [35,36,37,38]. Consequently, a threshold value of >25 Ncm was defined and applied in this study. Depending on the implant manufacturer, however, it is recommended not to exceed insertion torques of 50 Ncm, as higher values may lead to necrosis of the surrounding bone tissue and, consequently, to failed osseointegration [39,40]. Although the evidence on this matter is somewhat controversial, the authors chose this upper limit to minimize potential compressive trauma to the peri-implant bone [41]. This torque range is also cited as sufficient for immediate implantation and immediate loading protocols [34,42].

Optimal primary stability is therefore a key consideration in clinical practice [43,44]. In a clinical study, Barone et al. [39] demonstrated that insertion torque influences peri-implant bone stability. They reported that implants placed with a high insertion torque (≥ 50 Ncm) were associated with increased peri-implant bone remodeling and more pronounced buccal soft-tissue recession. Another study reported similar findings, indicating that implants inserted with an insertion torque below 50 Ncm exhibited less marginal bone loss and reduced soft-tissue recession [40]. In that study, the success rate was 98.2% for implants placed with a regular insertion torque, compared with 91.3% for those placed with a high insertion torque (≥ 50 Ncm).

The results of the present study, in line with our previous investigation, demonstrate that large implant diameters generate very high insertion torques (IT) in D1 to D3 bone. The null hypothesis (H_0_) can be rejected, as an adjustment of the drilling protocol is especially necessary in very dense (D1) and dense (D2) bone to achieve or maintain the desired torque range and thereby protect the peri-implant bone. As expected, overpreparation—defined as the use of the next larger drill diameter—leads to a significant reduction in insertion torque. This effect is further enhanced, albeit to a lesser extent, using a thread cutter (tap). These adjustments show favorable outcomes in dense bone (D2 = 25 PCF) and can therefore be recommended for use at this bone density. In very dense bone (D1 = 35 PCF), the combined use of the next larger drill diameter, a crestal drill, and a thread cutter appears advisable. However, for implants with very large diameters (>4.7 mm), additional measures may be necessary, as extremely high insertion torques (>80 Ncm) can still occur despite modifications to the drilling protocol. A potential clinical solution, as observed by the authors, is repeated insertion and removal of the implant to gradually widen the osteotomy. However, no data are currently available to support this approach. Investigation of this method may be a subject of future research.

Another clinical approach becomes relevant when the implant still protrudes more than 2 mm above the bone at an insertion torque of 35 Ncm. In such cases, placement of the implant at bone level with an acceptable torque is not feasible. If, upon reaching an insertion torque of 35 Ncm, the implant still requires advancement of more than 2 mm, it is necessary to remove the implant and further prepare the osteotomy using overpreparation and tap drilling. This allows for subsequent implant placement at bone level with an acceptable insertion torque.

When tapered implants are inserted into D1 and D2 bone using the preparation protocols applied in this study, slightly reduced insertion torque (IT) values can be expected. This suggests that in very dense bone, and when using a drill one size larger, tapered implants—especially those with larger diameters—tend to produce somewhat lower torque values compared to standard drilling protocols. With this implant geometry, excessive insertion torque in very dense bone may be avoided without necessarily having to modify the drilling protocol. Tapered implants were originally developed for use in low-density bone to achieve sufficient insertion torque in such conditions. Consequently, most studies on tapered implants focus on their performance in soft bone [45,46,47]. This implant type has been shown to achieve sufficient primary stability for immediate implantation and loading in low-density bone [45]. The tapered implant used in this study (SICtapered^®^) appears to function as a universal implant that can be used in both very dense and soft bone. It demonstrated the highest performance in terms of primary stability in soft bone, while interestingly not producing excessive insertion torques in dense bone.

In D2 bone, our results indicate that underpreparation was associated with higher primary stability for implants of smaller diameters, whereas this effect was not observed for larger diameters. This finding may suggest the existence of a diameter-dependent threshold beyond which further underpreparation does not yield additional benefits in terms of insertion torque. A possible explanation could be that, for larger diameters in medium-density bone, the contact surface and resulting friction may already be sufficient to achieve adequate stability, rendering additional underpreparation unnecessary or even potentially detrimental. Further investigations are warranted to determine whether such a threshold exists and to establish clinically relevant guidelines for protocol adjustment based on implant diameter.

In soft (D3 = 15 PCF) and very soft bone (D4 = 10 PCF), underpreparation protocols were applied in response to the bone quality. As expected, long implants with larger diameters also resulted in the greatest increase in insertion torque in these bone types—however, to a significantly lesser extent than in D1 and D2 bone. Despite the modifications, only a minimal increase in torque was observed for commonly used diameters of 3.7 mm and 4.2 mm, and the desired torque range was largely not achieved. In D3 bone, adjustments to the drilling protocol appear to have only limited effectiveness. Therefore, a more conservative approach may be advisable, and immediate loading should be critically evaluated or potentially avoided. This recommendation becomes even more relevant in very soft bone with a density of D4 (10 PCF), where nearly all insertion torque values remained below 25 Ncm—regardless of implant geometry, implant size, or any combination of drilling protocol modifications. In this bone quality, immediate implantation and immediate loading must be regarded as critical and should be approached with caution.

As an alternative to underpreparation of the implant site, the use of implants with aggressive thread designs or with an increased thread depth should also be considered, as these designs generate higher insertion torque values in soft bone compared to implants with standard threads [48]. However, the influence of this specific implant design parameter represents a separate research question and lies beyond the scope of the present study.

In addition, techniques aimed at increasing bone density should be considered, including Summers’ osteotomes and bone condensers. Summers’ osteotome technique [49] was introduced to enhance primary stability and to widen the edentulous ridge without removing bone, promoting the formation of a compacted layer at the bone–implant interface within cancellous bone [50]. However, it may be associated with surgical trauma and a risk of inadvertent fractures or bone displacement. Osseodensification, as described by Huwais and Meyer [51], offers a more controlled approach. A densifying bur compacts bone through rolling and sliding contact, with minimal plastic deformation. During osteotomy preparation, this compaction autografts bone, increases peri-implant bone density, and improves the mechanical stability of the implant [51]. A recent systematic review on osseodensification by Althobaiti et al. [52] reported that osseodensification drilling yielded higher ISQ values than conventional drilling protocols. In addition, implant sites prepared with densifying burs exhibited greater bone density compared with conventionally drilled sites.

The drilling protocol adjustment recommendations derived from the present results apply specifically to the implant system and implant types used in this study. While general trends may be inferred for systems from other manufacturers, each implant system has unique characteristics, and therefore a case-specific analysis should be conducted when in doubt. Moreover, accurately assessing bone quality and selecting the corresponding drilling protocol is a complex process that requires a high level of clinical experience and tactile sensitivity from the operator. Additionally, instrument wear (e.g., of drills) may create the false impression of dense bone, which could lead to an inappropriate choice of drilling protocol modification and, ultimately, the failure of immediate implantation and loading.

Preoperative bone density assessment may provide a valuable aid in this regard [53,54]. Toia et al. conducted a clinical study to evaluate insertion torque (IT) and marginal bone loss following drilling protocol modifications based on intraoperatively perceived bone quality [55]. They recommend initial assessment of bone density to ensure that the drilling protocol can be appropriately adjusted to avoid excessive insertion torque, which may negatively affect the marginal bone level. Similar to the findings of the present investigation, Toia et al. observed a significant influence of bone density and drilling protocol on insertion torque. Notably, they found that underpreparation was associated with significantly increased marginal bone loss around the implant. They concluded that both increasing bone density and higher insertion torque were correlated with greater marginal bone loss. These findings emphasize the importance of not exceeding a defined torque threshold to protect the peri-implant bone. Our results confirm that in very dense bone, overpreparation is often unavoidable to mitigate the effects described by Toia et al. [55]. This is relevant for implants with larger diameters, as in very dense (D1) and dense bone (D2), insertion torque values well above 50 Ncm can be reached with the standard drilling protocol. In this context, Gholami et al. [56] demonstrated in their clinical study that SICace implants, when placed using a modified drilling protocol, exhibit reduced crestal bone loss in D1 and D2 bone densities.

Since all implant types within the SIC system can be placed using the same surgical kit and drilling protocol, intraoperative adjustments to the drilling protocol based on the clinician’s tactile assessment of bone quality are readily feasible. After the initial pilot drilling, bone quality can typically be evaluated clinically, allowing for appropriate protocol adjustments before any overpreparation occurs. However, for less experienced clinicians or when drills become blunt due to wear, clinical assessment of bone quality—and thus correct adjustment of the drilling protocol—can be challenging.

Chrcanovic et al. [57], in their systematic review and meta-analysis, emphasized that preoperative assessment of bone quality in edentulous regions provides significant clinical and prognostic value. Poor bone quality and low density at the implant site are considered important risk factors that can negatively impact primary stability and osseointegration. Therefore, the assessment of bone density using cone beam computed tomography (CBCT) appears to be a reasonable approach. In medical applications, bone quality is typically expressed in Hounsfield units, whereas CBCT uses grayscale values, which are not direct measures of density and differ from Hounsfield units [58].

To achieve sufficient primary stability, clinicians must determine the appropriate drilling protocol and surgical approach prior to implant placement. However, identifying the optimal drilling protocol preoperatively remains a clinical challenge. The use of artificial intelligence (AI) and deep learning in implantology is growing rapidly [59,60]. Sakai et al. [61] developed a deep learning-based model aimed at predicting the optimal implant drilling protocol using CBCT images. Their results showed that deep learning offers a promising method for selecting an appropriate drilling protocol. Advances in AI could enable preoperative selection of optimal implant geometry and drilling protocol based on bone quality, allowing for even less experienced clinicians to place implants safely and predictably.

## 5. Conclusions

Adapted drilling protocols may enhance primary stability across bone densities, bridging the gap left by standard techniques. Clinicians should prioritize tapered implants in soft bone (D3) and consider protocol modifications to tailor surgical approaches to patient-specific bone quality. This strategy maximizes the success of immediate implant placement and immediate loading while minimizing complications.

In D1 bone, the drilling protocol should be adjusted by means of overpreparation to protect the native bone from compression necrosis. For large-diameter implants, a strategy to prevent excessive insertion torque must be defined prior to treatment. In D2 bone, smaller implant diameters generally require underpreparation, whereas larger implant bodies tend to reach the recommended torque range using the standard protocol. In D3 and D4 bone, the effects of adjusted drilling protocols are minimal and do not result in torque values sufficient to support immediate implantation and immediate loading. In these cases, a more conservative approach should be favored. These limitations highlight the need for further research into adjunctive techniques.

## Figures and Tables

**Table 1:** Implant types used in this study are presented with their corresponding diameters and lengths in millimeters (mm). SICace^®^ and SICmax^®^ feature a parallel-walled design, while SICtapered^®^ has a tapered implant geometry.

Implant Type	Implant Ø in mm	Implant Length in mm
SICace^®^	3.4	7.5
SICmax^®^	3.7	9.5
SICtapered^®^	3.7	13
SICace^®^	4.0	7.5
SICmax^®^	4.2	9.5
SICtapered^®^	4.2	13
SICace^®^	4.5	7.5
SICmax^®^	4.7	9.5
SICtapered^®^	4.7	13
SICace^®^	5.0	14.5
SICmax^®^	5.2	
SICtapered^®^	5.2	

**Table 2:** The four different bone qualities represented by polyurethane blocks corresponding to bone densities D1–D4 (D1 = PCF 35, D2 = 25, D3 = 15, D4 = 10). Depending on the bone quality, different drilling protocols were applied: O = oversized site preparation; U = undersized site preparation; S = standard preparation. The drilling sequence was adjusted by using the next larger or smaller drill, including or omitting the crestal drill, or using a tap drill. Additionally, the drilling depth was adjusted to 70% of the total drilling depth.

35 PCF (D1)			25 PCF (D2)			15 PCF (D3)				10 PCF (D4)		
O2	O1	S	O1	S	U1	S	S2	U1	U2	S	U1	U2

**Drilling Sequence**			**Last Drill**		**Drilling length**		**Crestal Drill**			**Tap Drill**		
S (Standard)			Standard		100%		✓			x		
S2 (Standard 2)			Standard		100%		x			x		
O1 (Oversized Site 1)			Next bigger drill		100%		✓			x		
O2 (Oversized Site 2)			Next bigger drill		100%		✓			✓		
U1 (Undersized Site 1)			Standard		70%		x			x		
U2 (Undersized Site 2)			Previous drill		100%		x			x		

## Data Availability

The original contributions presented in this study are included in the article. Further inquiries can be directed to the corresponding author.
